# Not All Transverse Sinus Stenoses Behave the Same: Distinct Flow Phenotypes on Phase-Contrast MRI

**DOI:** 10.7759/cureus.116366

**Published:** 2026-09-16

**Authors:** Erick D Villarreal-Ibañez, Juan C Mantilla-Suarez, Federico G. Lubinus-Badillo, C. Alejandro Luzardo-Neira, Juan C. Uribe-Caputti, Daniel E. Mantilla-Garcia

**Affiliations:** 1 Radiology, Fundación Oftalmológica de Santander – FOSCAL, Bucaramanga, COL; 2 Radiology, Universidad Autónoma de Bucaramanga, Bucaramanga, COL; 3 Radiology, Universidad Autonoma de Bucaramanga, Bucaramanga, COL; 4 Epidemiology and Public Health, Universidad Autónoma de Bucaramanga, Bucaramanga, COL; 5 Interventional Radiology, Fundación Oftalmológica de Santander – FOSCAL, Bucaramanga, COL; 6 Interventional Radiology, Universidad Autonoma de Bucaramanga, Bucaramanga, COL

**Keywords:** cerebral venous hemodynamics, idiopathic intracranial hypertension, intracranial venous flow, magnetic resonance venography, phase-contrast magnetic resonance imaging, transverse sinus stenosis

## Abstract

Background

Transverse sinus stenosis is usually assessed by its anatomical appearance, although stenoses with similar morphology may not have the same local flow behavior. This study evaluated peak velocity changes across transverse sinus stenoses using phase-contrast magnetic resonance imaging (MRI) and explored whether distinct hemodynamic patterns could be identified.

Methods

We retrospectively reviewed 183 consecutive patients who underwent brain MRI, magnetic resonance venography, and venous phase-contrast MRI for headache, vertigo, and/or tinnitus between September 2025 and June 2026. Transverse sinus stenosis was identified in 14 patients, including four with bilateral stenosis, yielding 18 stenotic segments. Peak velocity was measured immediately proximal to the stenosis, at the point of greatest narrowing, and immediately distal to it. Each segment was classified as Jet when intra-stenotic velocity exceeded both adjacent measurements, Low-flow when it was lower than both, or Intermediate when neither criterion was met.

Results

The mean age of the 14 patients was 50.7 ± 10.1 years, 11 (78.6%) were women, and the mean body mass index was 27.4 ± 3.5 kg/m². Headache was present in 10 patients (71.4%), vertigo in five (35.7%), and tinnitus in two (14.3%). Across all stenotic segments, mean peak velocity was 16.97 cm/s before the stenosis, 12.84 cm/s within it, and 14.82 cm/s after it. Seven segments (38.9%) showed a Jet pattern, with mean velocities of 14.26, 18.49, and 11.43 cm/s, respectively. Ten segments (55.6%) showed a Low-flow pattern, with corresponding velocities of 19.00, 8.92, and 18.14 cm/s. One segment (5.6%) had an Intermediate pattern. Five patients (35.7%) had three or more imaging findings suggestive of idiopathic intracranial hypertension.

Conclusions

Transverse sinus stenoses showed heterogeneous local flow behavior on phase-contrast MRI. Reduced intra-stenotic velocity was more common than the expected acceleration pattern, suggesting that anatomical narrowing does not represent a single hemodynamic entity. These preliminary phenotypes require validation against stenosis morphology, venous manometry, and clinical outcomes before their clinical relevance can be established.

## Introduction

Idiopathic intracranial hypertension (IIH) is characterized by raised intracranial pressure in the absence of an identifiable secondary cause. It predominantly affects women of childbearing age and is strongly associated with obesity. Headache and visual symptoms are the most common manifestations, and persistent intracranial hypertension may result in disabling headache and irreversible visual loss [1-3].

Diagnosis of IIH requires integration of clinical findings, neuroimaging, and cerebrospinal fluid (CSF) pressure measurements [2,4]. Magnetic resonance imaging (MRI) plays an essential role in excluding secondary causes and identifying imaging findings associated with IIH, including empty or partially empty sella, enlargement of the perioptic subarachnoid space, optic nerve tortuosity, posterior globe flattening, and transverse sinus stenosis (TSS) [4-6]. Among these findings, TSS is one of the most frequently reported imaging abnormalities in IIH and is strongly associated with the disorder [5,7]. However, the presence of TSS or other associated MRI findings alone does not establish a diagnosis of IIH, which requires appropriate clinical and, when indicated, ophthalmologic and CSF pressure assessment.

Despite the strong association between TSS and IIH, the pathophysiological and hemodynamic significance of TSS remains incompletely understood. Venographic and manometric studies have demonstrated pressure gradients across stenotic venous segments, while morphological studies have identified both intrinsic intraluminal abnormalities and extrinsic compression as potential mechanisms of stenosis [8-10]. These observations raise the possibility that TSS represents a heterogeneous group of anatomical and physiological conditions rather than a single pathological entity. Importantly, this heterogeneity may also be present in individuals with TSS who do not have clinically established IIH.

Current imaging assessment of TSS is predominantly anatomical. Magnetic resonance venography allows characterization of the location and morphology of luminal narrowing but provides limited information about its local hemodynamic consequences. Phase-contrast MRI (PC-MRI) enables noninvasive quantitative assessment of blood-flow velocity and therefore offers a potential approach for evaluating the functional behavior of stenotic venous segments [11]. More recently, 4D-flow MRI has been used to characterize the hemodynamics of TSS. Ding et al. demonstrated an association between trans-stenotic velocity differences and cerebral venous pressure gradients, while Bi et al. evaluated flow parameters at inflow, stenotic, and outflow planes to distinguish patients with high versus low trans-stenotic pressure gradients [12,13].

These studies establish that quantitative flow imaging can provide information beyond anatomical assessment; however, the specific patterns of local peak-velocity change across individual stenotic segments remain insufficiently characterized. In particular, whether TSS consistently exhibits intra-stenotic acceleration or whether distinct patterns of velocity increase, reduction, or intermediate behavior can be identified has not been adequately explored. From a conventional hemodynamic perspective, focal luminal narrowing is expected to increase local flow velocity as the effective cross-sectional area decreases [14]. Whether this conventional acceleration pattern applies uniformly to TSS remains unclear.

Therefore, this study aimed to characterize local flow velocity behavior across transverse sinus stenoses using PC-MRI by comparing peak velocities immediately proximal to, within, and distal to the stenotic segment and to explore whether distinct hemodynamic phenotypes could be identified. Because TSS is strongly associated with IIH, we additionally evaluated MRI findings suggestive of IIH and explored their relationship with venous flow measurements. The study population was not restricted to patients with clinically confirmed IIH; rather, it comprised patients with TSS identified on MR venography who underwent systematic assessment of associated imaging findings and local venous hemodynamics. We hypothesized that transverse sinus stenoses would demonstrate heterogeneous local flow behavior rather than a uniform pattern of intra-stenotic acceleration.

## Materials and methods

Study design and population

This retrospective observational study was conducted at a tertiary referral center specializing in neuroimaging. Consecutive patients referred for brain MRI between September 2025 and June 2026 because of headache, vertigo, and/or tinnitus were evaluated.

During the study period, 183 patients underwent brain MRI according to the same institutional imaging protocol. In all patients, the protocol included conventional brain MRI sequences, magnetic resonance venography (MRV), and venous PC-MRI for quantitative assessment of intracranial venous flow.

All examinations were evaluated for the presence of TSS. TSS was identified in 14 of the 183 patients (7.7%). These 14 patients comprised the study population and underwent the demographic, anthropometric, imaging, and hemodynamic analyses presented in this study. Four patients had bilateral TSS, resulting in a total of 18 stenotic transverse sinus segments available for hemodynamic evaluation.

The study was approved by the institutional ethics committee of Fundación Oftalmológica de Santander (FOSCAL). Given the retrospective nature of the analysis and the use of previously acquired imaging data, all procedures were conducted in accordance with institutional guidelines and the ethical principles of the Declaration of Helsinki.

Inclusion criteria

Patients were eligible for inclusion if they underwent brain MRI during the study period because of headache, vertigo, and/or tinnitus and demonstrated TSS on MRV. Patients were also required to have adequate phase-contrast MRI images for evaluation of venous flow velocities across the stenotic segment.

MRI acquisition protocol

All examinations were performed on a 3.0-T MRI system (Canon Medical Systems, Floridablanca, Santander, Colombia). Brain MRI was performed according to the institutional imaging protocol and included sagittal and axial T2-weighted fluid-attenuated inversion recovery (FLAIR), coronal and axial T2-weighted imaging, axial T1-weighted imaging, axial diffusion-weighted imaging (DWI) with corresponding apparent diffusion coefficient (ADC) maps, axial T2*-weighted gradient-echo imaging, and time-of-flight (TOF) MR venography.

Venous phase-contrast MRI was performed using an ECG-gated acquisition for quantitative assessment of intracranial venous flow. MR venography was used as the anatomical reference for planning the phase-contrast acquisitions. At each measurement location, the acquisition plane was individually adjusted according to the local course of the transverse sinus and positioned as perpendicular as possible to the vessel axis to minimize potential velocity measurement errors related to oblique flow. The acquisition parameters were as follows: repetition time (TR), 23.5 ms; echo time (TE), 10 ms; acquisition matrix, 256 × 256; velocity encoding (VENC), 20 cm/s; 16 cardiac phases; and 128 segments.

Peak velocity measurements were obtained at three predefined locations along each TSS: immediately proximal to the stenotic segment (pre-stenotic), at the point of maximal luminal narrowing (intra-stenotic), and immediately distal to the stenosis (post-stenotic). The acquisition plane was individually positioned at each of these three locations as perpendicular as possible to the local course of the transverse sinus. Regions of interest were placed over the entire cross-sectional lumen of the venous sinus at each location, and peak velocity values were recorded in centimeters per second (cm/s) for subsequent analysis.

Imaging analysis

All examinations were evaluated for TSS by a radiologist with approximately 20 years of experience in neuroradiology. TSS was identified on MRV as focal or segmental luminal narrowing relative to the adjacent venous sinus caliber. Stenosis severity was assessed morphologically but was not formally quantified as a percentage of luminal narrowing or categorized into severity grades. Contralateral transverse sinus hypoplasia was specifically assessed and recorded when present; however, transverse sinus dominance was not formally classified.

For each stenotic segment, three measurement locations were defined: the pre-stenotic segment, immediately proximal to the area of maximal narrowing; the intra-stenotic segment, corresponding to the point of maximal luminal narrowing; and the post-stenotic segment, immediately distal to the stenosis.

Phase-contrast MRI post-processing and quantitative velocity measurements were performed by a second reader with four years of experience in neuroimaging. At each measurement location, a region of interest (ROI) encompassing the entire cross-sectional lumen of the venous sinus was manually defined. Mean velocity was calculated across the ROI for each cardiac phase, and peak velocity was defined as the maximum mean velocity recorded across the cardiac cycle. Peak velocity values were recorded in centimeters per second (cm/s) at the MRI workstation. Representative examples of the acquisition plane, ROI placement, and velocity-time curve are shown in Figure 1.

**Figure 1:**
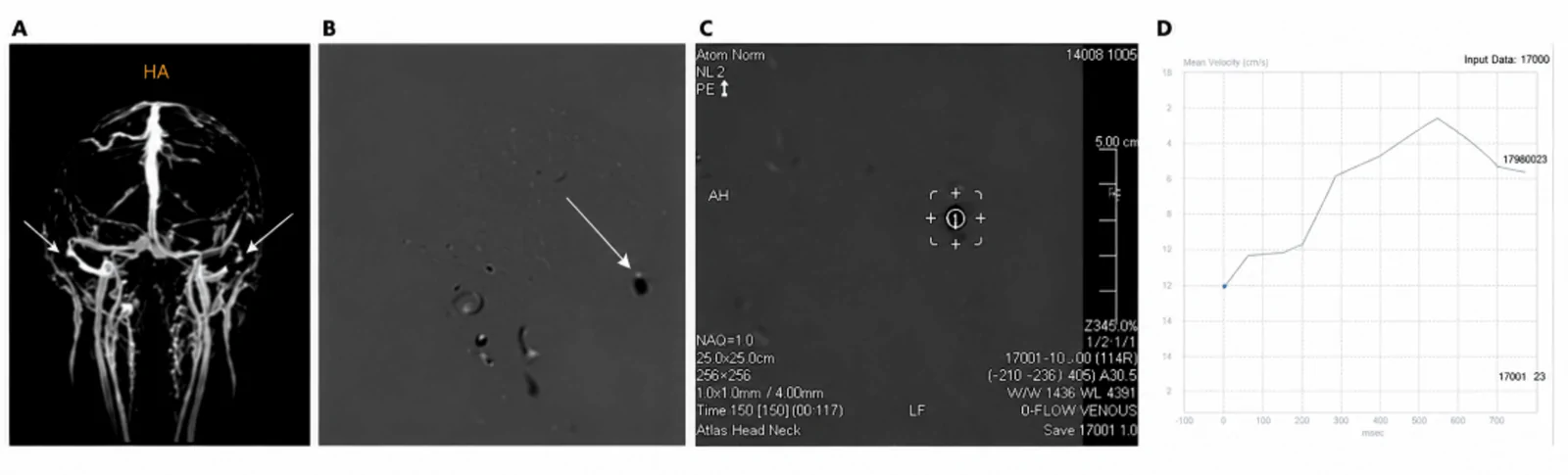
Phase-contrast MRI acquisition and velocity measurement in transverse sinus stenosis. (A) Magnetic resonance venography in a representative patient with bilateral transverse sinus stenosis (arrows). Venous flow measurements were obtained at three predefined locations for each stenotic segment: pre-stenotic, intra-stenotic, and post-stenotic. (B) Representative phase-contrast MRI acquisition used for venous flow assessment; the arrow indicates the transverse sinus. (C) Representative placement of the region of interest (ROI) encompassing the entire cross-sectional lumen of the venous sinus. (D) Representative velocity-time curve automatically generated by the MRI system during post-processing. The vertical axis represents mean velocity (cm/s), and the horizontal axis represents the cardiac cycle divided into 16 cardiac phases. Mean velocity was calculated across the ROI for each cardiac phase, and peak velocity was defined as the maximum mean velocity recorded across the cardiac cycle.

The pre-stenotic, intra-stenotic, and post-stenotic peak velocity measurements were subsequently used to characterize the hemodynamic behavior of each TSS.

Hemodynamic characterization

Each stenotic segment was classified according to the relationship between pre-stenotic, intra-stenotic, and post-stenotic peak velocities. Based on the observed velocity patterns, stenoses were classified into three exploratory hemodynamic phenotypes: (i) Jet phenotype: defined as an increase in intra-stenotic velocity relative to both the proximal and distal segments, consistent with trans-stenotic flow acceleration; (ii) Low-flow phenotype: defined as a decrease in intra-stenotic velocity relative to the adjacent segments, indicating flow deceleration within the stenosis; (iii) Intermediate phenotype: intra-stenotic velocity was intermediate between the pre-stenotic and post-stenotic velocities, without fulfilling the criteria for either the Jet or Low-flow phenotype.

This exploratory classification was developed to characterize the heterogeneous velocity patterns observed in the study population and was intended as a hypothesis-generating framework rather than a validated diagnostic classification system.

Statistical analysis

Continuous variables were summarized using means and standard deviations or medians and interquartile ranges, according to data distribution. Categorical variables were expressed as frequencies and percentages.

Flow velocities were analyzed separately for pre-stenotic, intra-stenotic, and post-stenotic segments. Descriptive analyses were performed for the entire cohort of stenotic segments and for each hemodynamic phenotype.

Given the exploratory nature of the study and the limited sample size, the primary focus was placed on descriptive characterization of velocity patterns rather than on inferential statistical comparisons. Statistical analyses were performed using Stata version 14 (StataCorp LLC, College Station, Texas, USA).

## Results

Among the 183 patients who underwent brain MRI during the study period, TSS was identified in 14 patients (7.7%). Four patients had bilateral stenosis, yielding a total of 18 stenotic transverse sinus segments for hemodynamic analysis.

The clinical and imaging characteristics of the study population are summarized in Table 1. The mean age was 50.7 ± 10.1 years, and the mean body mass index (BMI) was 27.4 ± 3.5 kg/m². The majority of patients were women (11/14, 78.6%). Headache was the most frequently reported symptom, present in 10 patients (71.4%), followed by vertigo in five (35.7%) and tinnitus in two (14.3%). Contralateral transverse sinus hypoplasia was identified in three patients (21.4%). Among the imaging findings suggestive of IIH, increased perioptic subarachnoid space was the most frequent, observed in six patients (42.9%), followed by empty sella and partially empty sella, each present in five patients (35.7%). Meckel's cave enlargement was identified in three patients (21.4%), whereas optic nerve tortuosity and posterior globe flattening were each present in one patient (7.1%).

**Table 1:** Clinical and imaging characteristics of patients with transverse sinus stenosis (n = 14). Values are presented as n (%). * mean ± SD. IIH, idiopathic intracranial hypertension.

Characteristic	n (%)
Demographic and anthropometric characteristics	
Age, years	50.7 ± 10.1*
Female sex	11 (78.6)
BMI, kg/m²	27.4 ± 3.5*
Clinical symptoms	
Headache	10 (71.4)
Vertigo	5 (35.7)
Tinnitus	2 (14.3)
Imaging findings	
Bilateral transverse sinus stenosis	4 (28.6)
Contralateral transverse sinus hypoplasia	3 (21.4)
Empty sella	5 (35.7)
Partially empty sella	5 (35.7)
Increased perioptic subarachnoid space	6 (42.9)
Meckel's cave enlargement	3 (21.4)
Optic nerve tortuosity	1 (7.1)
Posterior globe flattening	1 (7.1)
≥3 imaging findings suggestive of IIH	5 (35.7)

Five patients (35.7%) met the predefined imaging criterion of three or more findings suggestive of IIH. Within this subgroup, empty sella and increased perioptic subarachnoid space were present in all five patients (100%). Meckel's cave enlargement was present in three patients (60.0%), whereas posterior globe flattening and optic nerve tortuosity were each present in one patient (20.0%).

Flow velocities were measured proximal to, within, and distal to each stenotic segment using phase-contrast MRI. Across the 18 stenotic segments, mean peak velocity was 16.97 cm/s in the pre-stenotic segment, 12.84 cm/s in the intra-stenotic segment, and 14.82 cm/s in the post-stenotic segment.

Considerable variability was observed in the hemodynamic behavior of individual stenoses. While some lesions demonstrated the expected increase in velocity within the stenotic segment, others exhibited marked flow deceleration. The heterogeneous velocity patterns observed across stenotic segments prompted classification into three exploratory hemodynamic phenotypes (Figure 2).

**Figure 2:**
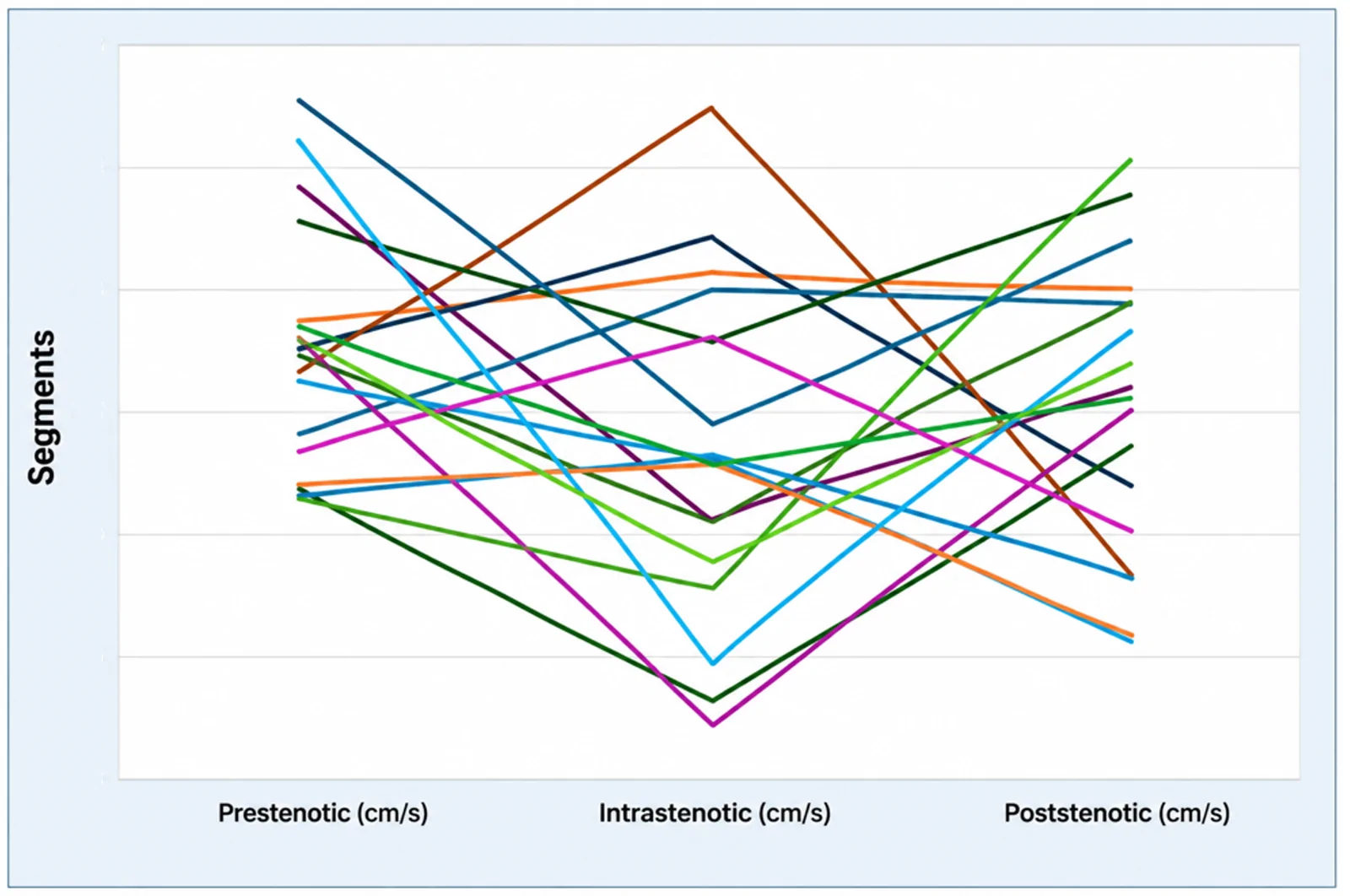
Flow velocity patterns across transverse sinus stenotic segments. Pre-stenotic, intra-stenotic, and post-stenotic peak velocities are shown for each of the 18 stenotic transverse sinus segments. Each color represents an individual stenotic segment and is used solely for visual differentiation and tracking across the three measurement locations; colors do not represent specific hemodynamic phenotypes or clinical categories.

Three distinct hemodynamic phenotypes were identified based on the predefined relationship between pre-stenotic, intra-stenotic, and post-stenotic velocities. The Jet phenotype was observed in seven of the 18 stenotic segments (38.9%), the Low-flow phenotype in 10 (55.6%), and the Intermediate phenotype in one segment (5.6%). The velocity characteristics of each phenotype are summarized in Table 2.

**Table 2:** Hemodynamic characteristics of transverse sinus stenoses according to flow phenotype. Values represent mean peak velocity for each phenotype. Velocities are reported in centimeters per second (cm/s).

Hemodynamic phenotype	Stenotic segments, n (%)	Pre-stenotic (cm/s)	Intra-stenotic (cm/s)	Post-stenotic (cm/s)
Jet	7 (38.9%)	14.26	18.49	11.43
Low-flow	10 (55.6%)	19.00	8.92	18.14
Intermediate	1 (5.6%)	15.7	12.6	5.3

The Jet phenotype was characterized by acceleration of blood flow within the stenotic segment, with intra-stenotic velocity exceeding both pre-stenotic and post-stenotic measurements. Mean velocities were 14.26 cm/s in the pre-stenotic segment, 18.49 cm/s within the stenosis, and 11.43 cm/s in the post-stenotic segment.

The Low-flow phenotype was the most frequent pattern, identified in 10 of the 18 stenotic segments (55.6%). In these lesions, intra-stenotic velocity was lower than both pre-stenotic and post-stenotic measurements. Mean velocities were 19.00 cm/s in the pre-stenotic segment, 8.92 cm/s within the stenosis, and 18.14 cm/s in the post-stenotic segment.

One stenotic segment (5.6%) demonstrated an Intermediate phenotype, with an intra-stenotic velocity that did not meet the predefined criteria for either the Jet or Low-flow phenotype. This segment showed velocities of 15.7 cm/s, 12.6 cm/s, and 5.3 cm/s in the pre-stenotic, intra-stenotic, and post-stenotic segments, respectively.

## Discussion

In this study, phase-contrast MRI demonstrated substantial hemodynamic heterogeneity among transverse sinus stenoses. Rather than exhibiting a uniform flow pattern, stenotic segments demonstrated three distinct hemodynamic phenotypes: a Jet phenotype characterized by intra-stenotic flow acceleration, a Low-flow phenotype characterized by reduced intra-stenotic velocity, and an Intermediate phenotype. Notably, the Low-flow phenotype was observed in 55.6% of stenotic segments, whereas the Jet phenotype was identified in 38.9%. These findings suggest that anatomical narrowing alone may not fully characterize the functional behavior of TSS. Importantly, these phenotypes should be considered exploratory and descriptive rather than validated pathophysiological entities.

Current understanding of TSS in IIH has largely been based on anatomical imaging. Most studies have characterized stenoses according to their presence, location, severity, or morphology, whereas relatively less attention has been directed toward their local hemodynamic behavior. From a conventional hemodynamic perspective, focal luminal narrowing is expected to increase local flow velocity as the effective cross-sectional area decreases [14]. However, this expected acceleration pattern was not observed uniformly in our cohort. One possible explanation is that velocity across a stenosis depends not only on luminal caliber but also on the volume of blood traversing the affected sinus. Redistribution of venous drainage toward the contralateral transverse sinus or collateral venous pathways could reduce flow through a stenotic segment and attenuate the expected velocity acceleration. Differences in venous anatomy and the functional severity of the stenosis may further contribute to the heterogeneous velocity behavior observed. These mechanisms remain hypothetical because volumetric flow, collateral venous drainage, and trans-stenotic pressure gradients were not systematically assessed in the present study.

The Jet phenotype, observed in approximately one-third of stenotic segments, was consistent with conventional hemodynamic expectations. In these lesions, velocity increased within the narrowed segment and subsequently decreased distally, resembling the behavior expected across focal flow-restricting lesions. This pattern could reflect a focal structural stenosis caused by an intraluminal abnormality, such as a septum or prominent arachnoid granulation, both of which have been described as potential anatomical substrates of transverse sinus narrowing [8,9]. Such lesions may behave as relatively fixed structural stenoses that concentrate flow through a reduced cross-sectional area, resulting in increased local velocity.

Notably, two of the six patients contributing Jet-pattern segments (33.3%) met the predefined imaging criterion of three or more findings suggestive of IIH. One patient had unilateral right TSS, whereas the other had bilateral TSS, with a Jet flow pattern in both stenotic segments. Together, these patients accounted for three of the seven Jet-pattern stenotic segments (42.9%). Both patients demonstrated an empty sella, increased perioptic subarachnoid space, and Meckel’s cave enlargement. This imaging-based threshold was used exclusively as an analytical approach to identify patients with a higher burden of MRI findings associated with IIH and should not be interpreted as establishing a clinical diagnosis of IIH. Although these findings are exploratory and do not establish an association between IIH and the Jet phenotype, the observation of this flow pattern in patients with multiple imaging features suggestive of IIH warrants further investigation in larger cohorts incorporating clinical, ophthalmologic, and CSF opening-pressure data.

In contrast, the Low-flow phenotype was the most frequent pattern in our cohort and was characterized by reduced velocity within the stenotic segment with subsequent distal recovery. Several lesions demonstrated marked reductions in intra-stenotic velocity relative to the corresponding proximal measurements. This pattern is not readily explained by the simple expectation of velocity acceleration across a fixed focal narrowing and may indicate the contribution of additional hemodynamic mechanisms. Previous 4D-flow MRI work has demonstrated that intracranial venous hemodynamics are influenced by cerebrospinal fluid pressure and intracranial morphology, supporting the concept that venous flow behavior may not be determined solely by luminal caliber [15].

One possible explanation is that some transverse sinus stenoses represent dynamic or partially collapsible lesions rather than fixed anatomical obstructions. Previous studies have proposed that elevated intracranial pressure may compress the compliant walls of the transverse sinus and contribute to extrinsic stenosis [10]. In such circumstances, the stenotic segment may not behave as a rigid conduit but rather as a partially collapsible structure with complex local flow dynamics. Complex flow phenomena, including flow jets, vortical flow, and turbulent flow, have also been described in TSS using 4D-flow MRI and computational modeling [12]. These phenomena could contribute to complex local velocity distributions and may partly explain deviations from the simple acceleration pattern observed in some stenotic segments.

Another possibility is that different morphological subtypes of stenosis correspond to different hemodynamic phenotypes. The distinction between intrinsic and extrinsic stenoses has been recognized in the venous sinus literature [10]. Intrinsic lesions, including those associated with arachnoid granulations or septations, may produce focal acceleration patterns such as those observed in the Jet phenotype [8,9]. Conversely, extrinsic or compressive stenoses could potentially produce different local flow patterns, including the reduced intra-stenotic velocities observed in our Low-flow phenotype. Although the present study was not designed to establish morphological-hemodynamic correlations, the observed heterogeneity raises the possibility that anatomically similar stenoses may exhibit different functional flow behaviors. This hypothesis should be tested in future studies incorporating systematic morphological characterization and invasive hemodynamic measurements.

In the present cohort, the observed hemodynamic behaviors were not significantly associated with the burden of imaging findings suggestive of IIH. Therefore, although TSS is strongly associated with IIH, our findings suggest that its local velocity behavior may not be determined solely by the presence of IIH-associated imaging abnormalities. Importantly, the present study was not designed to establish an association between these hemodynamic phenotypes and clinically confirmed IIH because systematic ophthalmologic evaluation and CSF opening-pressure measurements were not available. Further prospective studies incorporating clinically established IIH, CSF opening pressure, and trans-stenotic pressure gradients are needed to determine whether these hemodynamic phenotypes have clinical relevance in IIH.

These findings may have implications for the interpretation of venous sinus imaging and for future studies evaluating endovascular treatment. Venous sinus stenting is used in selected patients with medically refractory IIH and significant trans-stenotic pressure gradients, although clinical response and pressure gradients vary across patients [10,16]. It is conceivable that different hemodynamic phenotypes may be associated with different underlying physiological conditions and, consequently, could have different implications for therapeutic response [12]. Hemodynamic characterization may ultimately complement anatomical assessment when evaluating patients with TSS. Importantly, however, the present study does not establish distinct underlying pathophysiological mechanisms or demonstrate that any specific phenotype predicts response to venous sinus stenting.

The present study has several strengths. To our knowledge, few studies have characterized local velocity behavior across transverse sinus stenoses using phase-contrast or 4D-flow MRI. Prior work has demonstrated that trans-stenotic velocity differences measured with 4D-flow MRI correlate with cerebral venous pressure gradients in patients with TSS [12], while more recent work has examined the relationship between cerebrospinal fluid pressure, intracranial morphology, and venous hemodynamics [15]. Our study extends this literature by evaluating velocity immediately proximal to, within, and distal to individual stenotic segments, thereby allowing characterization of local flow behavior rather than relying solely on global venous flow metrics. Furthermore, the identification of exploratory hemodynamic phenotypes provides a potential framework for describing venous sinus stenosis beyond conventional anatomical classifications.

Several limitations should be acknowledged. First, the sample size was relatively small, reflecting the exploratory nature of the study. Second, the retrospective design introduces the possibility of selection bias. Third, invasive venous manometry was not available, preventing correlation between the observed hemodynamic phenotypes and trans-stenotic pressure gradients. Fourth, morphological classification of stenoses into intrinsic and extrinsic subtypes was not systematically performed, limiting direct assessment of anatomical-hemodynamic relationships. Fifth, transverse sinus dominance was not formally classified, although contralateral transverse sinus hypoplasia was recorded when present. Anatomical asymmetry and redistribution of venous drainage could therefore have influenced local velocity measurements. Finally, phase-contrast MRI measurements are subject to technical factors that may influence local velocity estimation, particularly within focally narrowed venous segments. Partial-volume averaging and spatial resolution may affect measurements within small stenotic lumens, while deviations from a perfectly perpendicular acquisition plane may alter measured velocities. Although the acquisition plane was individually planned using MRV and positioned as perpendicular as possible to the local course of the transverse sinus at each measurement location, residual measurement variability cannot be excluded. In addition, phase-contrast MRI may not fully characterize complex three-dimensional flow phenomena occurring within stenotic venous segments. These considerations are particularly relevant when interpreting the reduced intra-stenotic velocities observed in the Low-flow phenotype.

Future investigations should evaluate larger prospective cohorts and incorporate detailed morphological characterization, venous manometry, assessment of venous drainage anatomy, and advanced flow imaging techniques. Correlation of anatomical subtype, trans-stenotic pressure gradient, and hemodynamic phenotype will be necessary to determine whether the distinct flow behaviors identified in this study represent reproducible physiological patterns and whether they have clinical significance. Such work could contribute to a more refined functional characterization of TSS and potentially complement conventional anatomical assessment in the evaluation of patients with venous sinus disease.

## Conclusions

Transverse sinus stenoses demonstrated substantial hemodynamic heterogeneity on phase-contrast MRI. Although some stenotic segments exhibited the expected pattern of intra-stenotic flow acceleration, the most frequent phenotype was characterized by reduced intra-stenotic velocity, observed in more than half of the stenotic segments. These findings suggest that transverse sinus stenosis may not represent a single hemodynamic entity and support the presence of distinct hemodynamic phenotypes characterized by different local velocity behaviors. These phenotypes should be considered exploratory and hypothesis-generating rather than established pathophysiological entities. Further studies integrating systematic morphological characterization, quantitative hemodynamic assessment, and venous manometry are warranted to validate these phenotypes and determine their physiological and potential clinical significance.
